# Functional evaluation of LTR-derived lncRNAs in porcine oocytes and zygotes with RNA-seq and small RNA-seq

**DOI:** 10.3389/fgene.2022.1023041

**Published:** 2022-10-13

**Authors:** Xu Yang, Jingzhang Ji, Hongdi Cui, Qi Zhao, Chunming Ding, Chang Xu

**Affiliations:** ^1^ School of Laboratory Medicine and Life Sciences, Wenzhou Medical University, Wenzhou, Zhejiang, China; ^2^ Oujiang Laboratory, Zhejiang Lab for Regenerative Medicine, Vision and Brain Health, Wenzhou Medical University, Wenzhou, Zhejiang, China; ^3^ Department of Colorectal Surgery, The First Affiliated Hospital of Wenzhou Medical University, Wenzhou, Zhejiang, China

**Keywords:** pig, lncRNA, LTR, embryo, RNA-seq, small RNA-seq

## Abstract

Long noncoding RNAs (lncRNAs) are increasingly being recognized as modulators of early embryonic development in mammals. However, they are seldom investigated in pigs. Here, to annotate full-length RNA transcripts, we performed annotation using a newly developed computational pipeline—an RNA-seq and small RNA-seq combined strategy—using our previously obtained RNA-seq and small RNA-seq data from porcine oocytes and zygotes. As evidenced by the length comparison, the frequency of the core promoter, and the polyadenylation signal motifs, the transcripts appear to be full-length. Furthermore, our strategy allowed the identification of a large number of endogenous retrovirus-associated lncRNAs (ERV-lncRNAs) and found that some of them were highly expressed in porcine zygotes, as compared to oocytes. Through the knockdown strategy, two ERV-lncRNAs (TCONS_00035465 and TCONS_00031520) were identified as playing potential roles in the early embryo development of pigs, laying a foundation for future research.

## Introduction

Long noncoding RNA (lncRNA) is a genomic transcription product of more than 200 nt with no or very little protein-coding ability (Jarroux et al., 2017; Ransohoff et al., 2018). lncRNAs can be divided into intragenic lncRNAs and intergenic lncRNAs, according to their derivation (Bouckenheimer et al., 2016). In mice and humans, more than two-thirds of lncRNAs are endogenous retrovirus- (ERV-) associated (Wang et al., 2016; Ransohoff et al., 2018). In addition, the LTR regions of ERVs enriched with pluripotency-related lncRNAs, such as the human pluripotency associated transcripts (HPATs), are involved in the formation of blastocyst ICMs and the maintenance of pluripotency (Durruthy-Durruthy et al., 2016). These findings point to likely roles for ERV-associated lncRNAs in the maintenance of pluripotency and lineage commitment. However, the functions of ERV-associated lncRNAs are difficult to evaluate due to their very low expression levels.

The development of early mammalian embryos, from zygote to blastocyst, is a strictly regulated process (Jukam et al., 2017). lncRNAs have been proven to participate in a wide variety of developmental stages. For example, we have reported that an ERV-associated lncRNA, lincGET, is identified as one of the earliest known lineage regulators to bias cell fate in the two-cell embryo, by promoting the nuclear localization of CARM1, and is essential for embryo development (Wang et al., 2016). However, almost all studies have been conducted in mouse embryos (Hamazaki et al., 2015; Wang et al., 2016; Wang et al., 2018; Geng et al., 2022). Given the poor conservation of ERV-associated lncRNAs among different species, it is essential to demonstrate their functional roles in the early embryos of other mammals. Pig, as an important livestock animal that shares conserved principles of early development with humans, is considered a human disease model and a potential organ donor for xenotransplantation in regenerative medicine (Platt, 1998; Lu et al., 2019; Zhou et al., 2022). Thus, the investigation of transcription element- (TEs-) associated lncRNAs in porcine early embryos can provide clues for understanding the early embryonic development of humans.

With the development of RNA sequencing (RNA-seq) technology, *de novo* transcripts annotation is becoming easier and easier (Reuter et al., 2015; Van Borm et al., 2015). However, due to the inevitable degradation of RNA during library building, annotation is unable to reach the 5′ ends of genes, especially the low-expressed ones; this constrains the functional annotation of lncRNAs. Our teams developed a computational pipeline, RSCS, to assemble RNA-seq and small RNA-seq data. This study raises the exciting possibility that the combination of RNA-seq and sRNA-seq could allow the determination of the 5′ and 3′ ends of low-expressed transcripts from small samples, such as from early embryos.

In this study, we combined our previous RNA-seq and sRNA-seq data on mature porcine oocytes and zygotes, respectively, to identify lncRNAs. We identified thousands of novel lncRNAs, and most of them seem to be exhibited full-length, with TSS and terminators. We also characterized a large number of LTR-derived lncRNAs (LTR-lncRNAs) with 5′ ends located in specific LTR retrotransposon families and identified two candidates that are essential for the early development of pigs.

## Materials and methods

### Porcine embryo culture and collection

All experiments were performed according to the guidelines of the State Key Laboratory Animal Care and Use Committee. The procedure for porcine IVF has been described previously (Kong et al., 2020). Briefly, freshly ejaculated sperm-rich fractions were collected from fertile boars. Following a short incubation at 39°C, semen was resuspended and washed three times in DPBS supplemented with 0.1% (w/v) BSA, then centrifuged at 1,500 g for 4 min. A hemocytometer was used to measure spermatozoan concentrations and to determine the proportion of motile sperm. Next, spermatozoa were diluted with modified tris-buffered medium (mTBM) to an optimal concentration. Cumulus-free oocytes were washed three times in mTBM. Approximately 30 oocytes were inseminated in 50 ml mTBM at a final sperm concentration of 300,000/ml for 5 h. Embryos were cultured in porcine zygote medium-3 (PZM-3) at 39°C in 5% CO_2_ in air. Embryos were collected after IVF at the following time points: one-cell stage (24 h), two-cell stage (40–45 h), four-cell stage (65–72 h), eight-cell stage (84–90 h), morula stage (108–115 h), and blastocyst stage (156–160 h). Additionally, the oocytes were collected after 42 h *in vitro* maturation. For qPCR, about 50 embryos of each stage were used. Embryo development was then observed every 24 h.

### Microinjection

To knock down lncRNAs, we injected about 10 pl 10 μM siRNA targeting lncRNAs into each MII oocyte and then performed IVF to obtain embryos. The sequences of siRNAs are shown in Table 1.

**TABLE 1:** List of major primers and siRNAs.

Primer name	Sequence (5′-3′)
TCONS_00035465-F	TCC​ACT​CCT​CTC​CAC​TTC​TGC​CA
TCONS_00035465-R	TCC​TAA​CAC​CGA​TAA​ACT​GCT​CC
TCONS_00031520-F	TCCACTTCTGCCACC
TCONS_00031520-R	TCCTAACACCGATAAACT
TCONS_00016256-siRNA-F	CCA​UCC​UCU​GCU​ACC​ACA​UTT
TCONS_00016256-siRNA-R	AUG​UGG​UAG​CAG​AGG​AUG​GTT
TCONS_00035465-siRNA-F	GGA​GCA​CAC​AAA​UUG​UCU​UTT
TCONS_00035465-siRNA-R	AAG​ACA​AUU​UGU​GUG​CUC​CTT
TCONS_00031520-siRNA-F	CCA​CCA​GAC​AAG​UGA​CUA​ATT
TCONS_00031520-siRNA-R	UUA​GUC​ACU​UGU​CUG​GUG​GTT
TCONS_00087790-siRNA-F	GCG​CCC​AAU​UCA​ACG​AUA​UTT
TCONS_00087790-siRNA-R	AUA​UCG​UUG​AAU​UGG​GCG​CTT
TCONS_00118,633-siRNA-F	CCG​CAA​GUG​GUG​AUG​CCA​ATT
TCONS_00118,633-siRNA-R	UUG​GCA​UCA​CCA​CUU​GCG​GTT
TCONS_00051895-siRNA-F	GCC​AUU​AGA​AUA​CCA​UCU​UTT
TCONS_00051895-siRNA-R	AAG​AUG​GUA​UUC​UAA​UGG​CTT
TCONS_00093469-siRNA-F	CCA​GCA​GUA​UGU​GCU​CCA​ATT
TCONS_00093469-siRNA-R	UUG​GAG​CAC​AUA​CUG​CUG​GTT
TCONS_00009915-siRNA-F	GCU​UGA​AGG​CAG​GUG​AUA​UTT
TCONS_00009915-siRNA-R	AUA​UCA​CCU​GCC​UUC​AAG​CTT
TCONS_00103,406-siRNA-F	GCU​UGG​CUC​ACA​CCU​UAA​UTT
TCONS_00103,406-siRNA-R	AUU​AAG​GUG​UGA​GCC​AAG​CTT
TCONS_00005047-siRNA-F	GCA​GAG​CUU​CUG​UAG​CAU​ATT
TCONS_00005047-siRNA-R	UAU​GCU​ACA​GAA​GCU​CUG​CTT
TCONS_00053217-siRNA-F	GCU​CCA​AGG​AUU​ACU​CAA​UTT
TCONS_00053217-siRNA-R	AUU​GAG​UAA​UCC​UUG​GAG​CTT
TCONS_00036389-siRNA-F	GCU​CCA​AAG​CGU​CCU​GGA​UTT
TCONS_00036389-siRNA-R	AUC​CAG​GAC​GCU​UUG​GAG​CTT
TCONS_00043789-siRNA-F	GCC​AUA​GAC​AGU​GGU​UCC​UTT
TCONS_00043789-siRNA-R	AGG​AAC​CAC​UGU​CUA​UGG​CTT
TCONS_00065478-siRNA-F	GGA​UGG​CAA​ACU​UAU​GCU​UTT
TCONS_00065478-siRNA-R	AAG​CAU​AAG​UUU​GCC​AUC​CTT
TCONS_00030592-siRNA-F	GGC​UAC​UAC​UUA​AGC​AUU​UTT
TCONS_00030592-siRNA-R	AAA​UGC​UUA​AGU​AGU​AGC​CTT
TCONS_0002148-siRNA-F	GGA​UUC​AGU​CCA​UGC​AUU​UTT
TCONS_0002148-siRNA-R	AAA​UGC​AUG​GAC​UGA​AUC​CTT
TCONS_00078948-siRNA-F	GCC​UAU​AAG​CCC​AAA​UUA​UTT
TCONS_00078948-siRNA-R	AUA​AUU​UGG​GCU​UAU​AGG​CTT
TCONS_00029836-siRNA-F	CCA​UCA​GAC​GGA​ACC​UCA​ATT
TCONS_00029836-siRNA-R	UUG​AGG​UUC​CGU​CUG​AUG​GTT
TCONS_00130,419-siRNA-F	GCA​GAG​UGG​AUG​CAU​GUA​UTT
TCONS_00130,419-siRNA-R	AUA​CAU​GCA​UCC​ACU​CUG​CTT
18S-F	CCC​GAA​GCG​TTT​ACT​TTG​A
18S-R	ACTTTGGTTTCCCGGAAG

### Real-time RT-PCR (qPCR) analysis

Total RNA was extracted using the PureLink Micro-to-Midi System (Invitrogen) according to the manufacturer’s instructions, and reverse transcription was used to generate cDNAs using the PrimeScript RT Reagent kit (TaKaRa). qPCR was performed using SYBR Premix Ex Taq (TaKaRa) and the 7,500 Real-Time PCR System. The reaction parameters were 95°C for 30 s, followed by 40 two-step cycles of 95°C for 5 s and 60°C for 34 s. Ct values were calculated using Sequence Detection System software, and the amount of target sequence normalized to the reference sequence was calculated as 2^−ΔΔCt^. All primers and probes were designed using Primer Premier 5.

### The RSCS pipeline

The RSCS pipeline includes 1) pre-configuration of sample data, 2) modified transcriptome assembly, and 3) transcript classification and lncRNA prediction.

#### Pre-configuration of sample data

The raw sequence data, in FASTQ format, were filtered to remove reads with unknown nucleotides, and FastQC (v0.11.5) was used for Illumina reads. Subsequently, Trim Galore (v0.6.4) software was used to discard low-quality reads, trim the adaptor sequences, and eliminate poor-quality bases. Specifically, Trim Galore discarded trimmed reads from the small RNA-seq data with less than 18 nucleotides or more than 50 nucleotides (parameter: “--small_rna --length 18”). RNA-seq and small RNA reads were mapped to 10 mm using HISAT2 (v2.1.0), and the unique reads were used in further analyses.

#### Modified transcriptome assembly

We then transformed Sequence Alignment/Map- (SAM-) format files to BAM format (a compressed binary version of the SAM format), discarded reads that were not aligned to the reference genome, and sorted the BAM file with SAMtools (v1.9).

For deep annotation, we considered the following features:

• BAM files are generated after alignment and after low-quality mapped reads have been filtered out.
• BAM is the compressed binary version of the SAM format, uses Blocked GNU Zip Format (BGZF) compression, and can support indexes to achieve fast random access by generating BAM index (BAI) files.

The BAM files of RNA-seq and small RNA-seq data obtained at identical biological stages were merged using SAMtools merge, generating a pooled BAM file. Importantly, this study used only reads with MAPQ values over 10 and that mapped to a single locus in the genomes. These steps were performed simultaneously with duplicate marking by running Mark Duplicates on all read groups in each pooled BAM file obtained for a sample; this procedure was repeated for each sample. We then ran base recalibration on the aggregated per-sample BAM files.

Transcript assemblies were performed separately for each sample using StringTie (v1.2.3) (Pertea et al., 2016) with GENCODE annotation (vM20) (Harrow et al., 2006) as a guide. The expression level of each gene was quantified by normalized FPKM (fragments per kilobase of exon per million mapped fragments) using StringTie. For new transcripts, data normalization was performed by transforming summational mapped transcript reads to RPKM.

#### Transcript classification and lncRNA prediction

We used StringTie merge to merge all the transcripts identified by the RSCS across all stages. The transcripts were annotated by comparison with mouse GENCODE annotation (vM20) using Cuffcompare (v2.2.1). The long-read transcripts were classified into four classes according to their most closely matching GENCODE transcript: “ = ” for “Exact match to annotation,” “e” and “j” for “Potentially novel isoform,” “u” for “Potentially novel gene,” and “Other.”

To identify reliable, multi-exonic long non-coding transcripts, we implemented the following selection criteria: 1) read coverage ≥3 in at least one of the tissues, 2) ≥200 bp, 3) ≥2 exons, 4) low protein-coding potential predicted by CPC2 and CPAT, and 5) no sense overlap with known coding genes derived from the UCSC, Ensembl, or Resfeq databases.

### Annotation of LTR-lncRNAs

Coordinates and annotations of TE elements were downloaded from the UCSC Genome Browser (7/3/2012 version of RepeatMasker). lncRNAs overlapping with TE elements were identified as TE-lncRNAs using BEDTools software. ERV-lncRNAs were further identified by RepeatMasker annotation. ERV-lncRNAs with expression levels (FPKM) > 1 were used for further analysis. An ERV-lncRNA PCA plot was performed in the R language.

### Statistical analysis

The statistical analyses were performed using R4.0.2. The data are shown as the means ± SEMs. Differences between the results obtained for two groups were evaluated using either two-tailed Student’s *t*-test or the Wilcoxon rank-sum test. The asterisks indicate significant differences: *p < 0.05, **p < 0.01, and ***p < 0.001.

## Results

### Annotation of transcripts by combined RNA-seq and sRNA-seq data in porcine oocytes and zygotes

**FIGURE 1:**
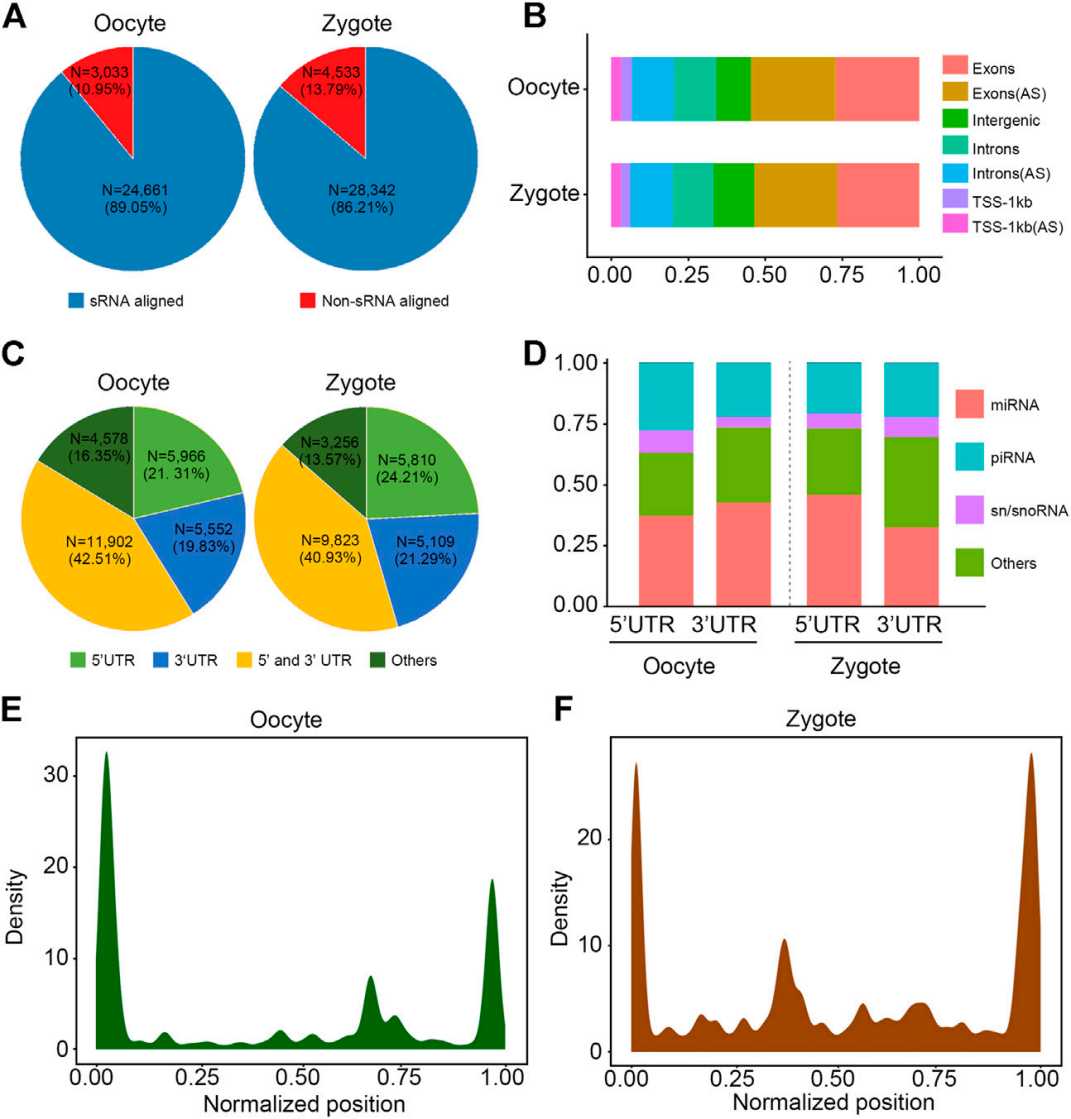
Annotation of transcripts by combination of RNA-seq and sRNA-seq data. **(A)** Pie chart shows that sRNA is involved in splicing in 89.05% and 86.21% of transcripts in porcine oocytes and zygotes, respectively. **(B)** Percentage of locations where sRNA data contributes. AS: antisense strand. **(C)** Pie chart shows that over 75% of the sRNA tags were positioned to the 3′ UTR and 5′ UTR of transcripts. UTR: untranslated regions. **(D)** Percentage of different classes of small RNA constituted over the 3′ UTR and 5′ UTR. **(E,F)** The density distribution of sRNA shows that most sRNA tags were positioned to the 3′ UTR and 5′ UTR of transcripts.

To comprehensively recognize the transcripts expressed in porcine oocytes and zygotes, we created extensive transcriptome profiles, based on million-tag sequences mapping to the reference genome from RNA-seq and small RNA-seq (sRNA-seq) (Table 2). In total, 27,694 and 32,875 high-quality non-redundant transcripts were assembled in oocytes and zygotes, respectively, and sRNA data was involved in the assembly of most transcripts (89.05% in oocytes and 86.21% in zygotes, Figure 1A). Furthermore, more than 50% of sRNA tags contributed to the exons in both oocytes and zygotes, although some tags contributed to the antisense chains (Figure 1B). Most of the sRNA tags contributing to the exons were positioned at the transcript terminals (23,402 vs 4,578 in oocytes and 20,702 vs 3,256 in zygotes, Figure 1C). microRNA constituted most of the terminal small RNA tags (Figure 1D). The density distribution of sRNA showed the same result (Figures 1E,F). Taken together, RNA-seq combined with sRNA-seq could allow for the determination of the 5′ and 3′ ends of low-expressed transcripts in pig oocytes and zygotes.

**TABLE 2:** Aligned tags of RNA-seq and short RNA-seq.

Aligned tags (x 10^6^)		
Samples	RNA-seq	Short RNA-seq
oocyte	131.32	14.42
zygote	184.47	9.59

### Transcripts annotated with small RNAs appear full-length

**FIGURE 2:**
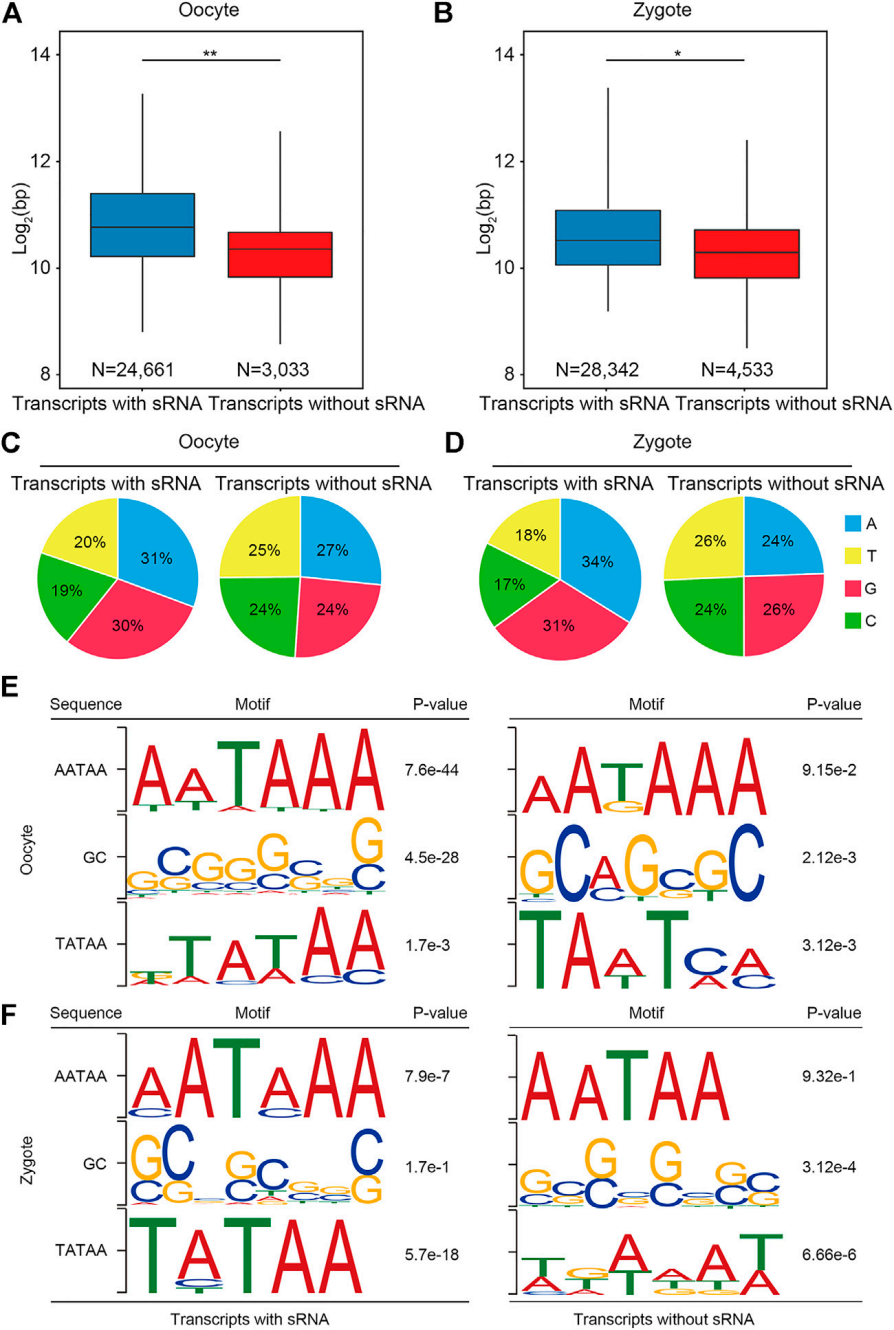
Annotation with small RNAs can produce a more complete transcript. **(A,B)** Box plot shows that the length of transcripts with sRNA and transcripts without sRNA in porcine oocytes and zygotes, respectively. **(C,D)** Pie chart shows that the first base for more than 60% of transcripts with sRNA was a purine base in both oocytes and zygotes. **(E,F)** Frequently observed sequence motifs upstream of the initial and end bases of the transcripts. The region used for motif identification and the *p* value are shown.

To further verify whether the transcripts obtained by RSCS were full-length transcripts, we analyzed the characteristics of the transcripts we obtained. Based on the above results (Figure 1A), transcripts could be divided into two classes: transcripts with sRNA and transcripts without sRNA. First, the lengths of the two classes of transcripts were statistically analyzed in both oocytes and zygotes (Figures 2A,B). We found that transcript length was significantly greater for transcripts with sRNA than for transcripts without sRNA, indicating that more complete RNA sequences were obtained with sRNA aligned. It has been shown that the first base of complete transcripts biases the purine bases (A and G) (Zecherle et al., 1996; Albersmeier et al., 2017). The first bases of the two classes of transcripts were also detected. The result shows that for more than 60% of transcripts with sRNA, the first base was A or G in both oocytes and zygotes, but this number was only about 50% for transcripts without sRNA in both oocytes and zygotes (Figures 2C,D). Furthermore, it has been reported that a complete transcript should have the transcription initiation sequence TATAA at the 5′ region and the transcription termination sequence AATAA and GC at the 3′ region (Gregor et al., 1986; Huck et al., 1986; Savinkova et al., 2009). Therefore, motif prediction was performed on the 5′ and 3′ regions of oocytes and zygotes. As expected, transcripts with sRNA showed clearer TATAA, AATAAA, and GC motifs than did transcripts without sRNA (Figures 2E,F). Taken together, these data demonstrate that transcripts annotated with small RNAs appear to be full-length.

### Characterization of lncRNAs in porcine oocytes and zygotes

**FIGURE 3:**
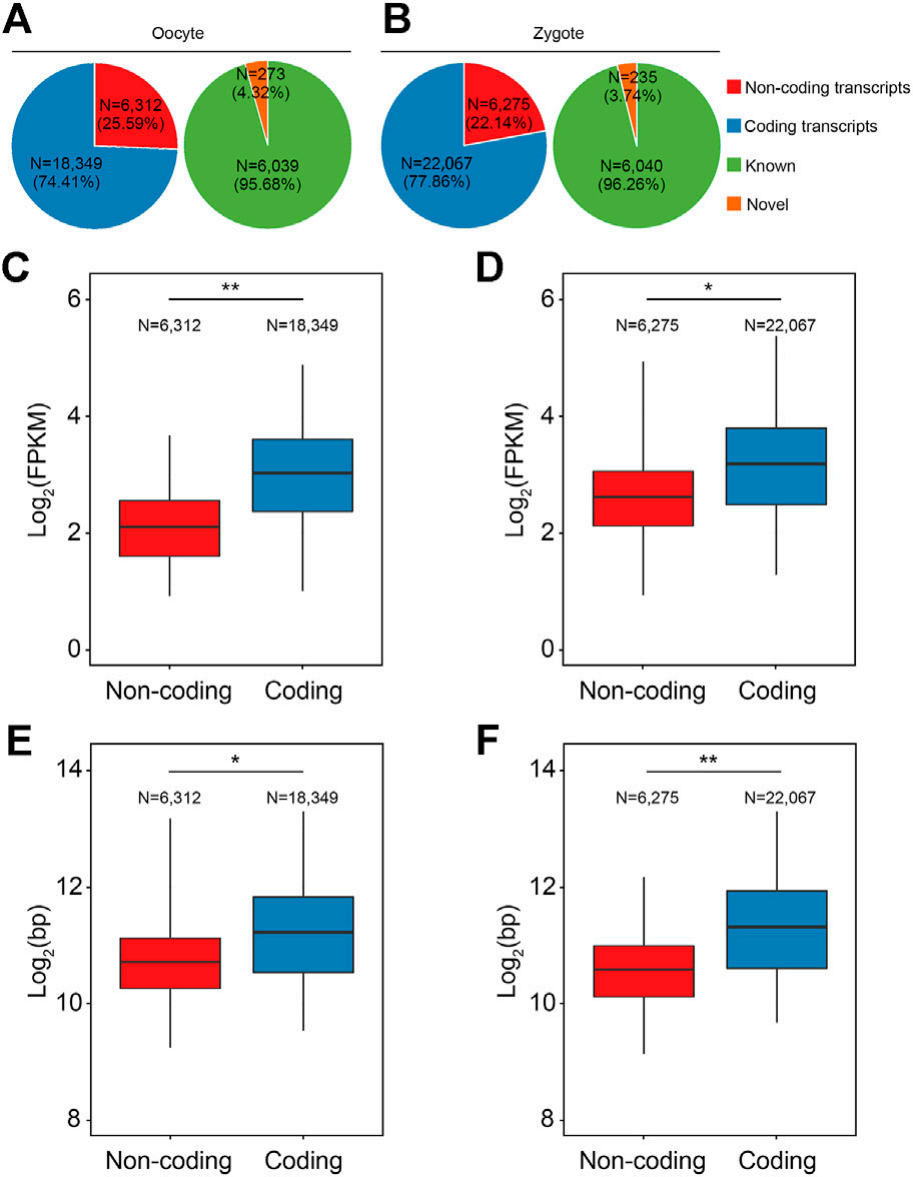
Characterization of lncRNAs in porcine oocytes and zygotes. **(A,B)** Pie chart shows the classification of the annotated and novel RSCS transcripts according to the GENCODE annotation or protein-coding potential. **(C,D)** Box plot shows that the expression level of the lncRNAs in pig were lower than in the coding genes. **(E,F)** Box plot shows that the transcript length of the lncRNAs in pigs was shorter than in the coding genes.

Next, transcripts with sRNA were divided into two classes: coding transcripts and non-coding transcripts, according to the GENCODE annotation and protein-coding potential of the novel transcripts using CPAT (v2.2.0) and CPC2 (v2.0). In the non-coding transcripts of oocytes, 4.32% were annotated and 95.68% were unannotated; in the non-coding transcripts of zygotes, 3.74% were annotated and 96.26% were unannotated (Figures 3A,B). Consistent with lncRNAs identified in mice and humans, in pigs the transcript lengths and expression levels of the lncRNAs were shorter and lower than those of coding genes (Figures 3C–F).

### Characterization of LTR-lncRNAs in porcine oocytes and zygotes

**FIGURE 4:**
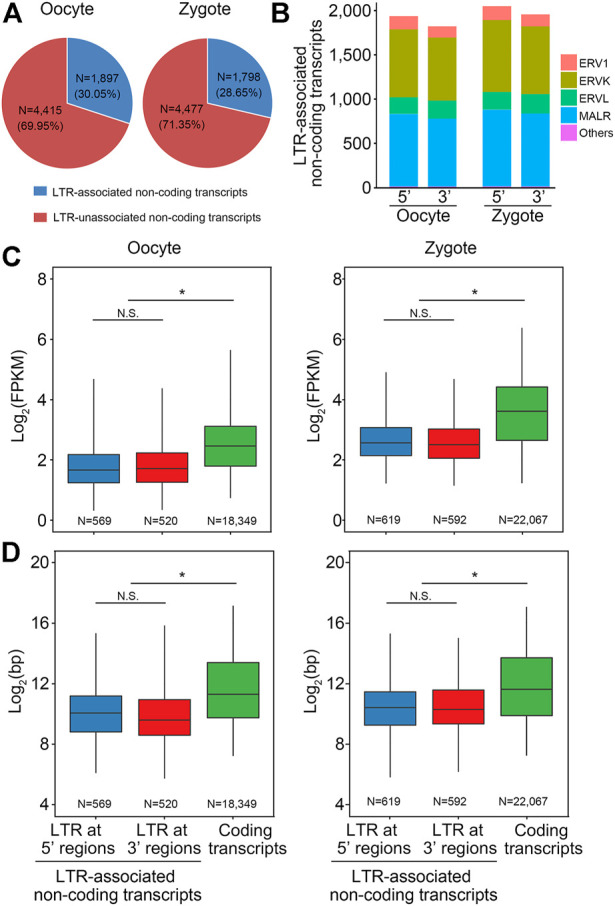
Characterization of LTR-lncRNAs in pigs. **(A)** Pie chart shows that about 30% lncRNAs are derived from LTR in porcine oocytes and zygotes. **(B)** LTR-associated transcripts in oocytes and zygotes were all from the ERV1, ERVK, ERVL, and MALR families, and most of the transcripts were from the endogenous retrovirus families ERVK and MALR, ERV: endogenous retrovirus. **(C)** Box plot shows that the expression level of the LTR-associated non-coding transcripts in pig was lower than the coding transcripts. **(D)** Box plot shows that the transcript length of the LTR-associated non-coding transcripts in pig was shorter than the coding transcripts.

Recent studies have revealed that families of ERV-associated lncRNAs are closely related to pluripotency; that the transcription of MERVL and HERVH or HERVK is a hallmark of two-cell embryo-like totipotent mouse embryonic stem cells (ESCs) and naïve-like human ESCs, respectively; and that LTR is an important regulatory region in ERV. To explore the regulatory mechanism of lncRNA on early embryo development in pigs, lncRNA derived from LTR was investigated in both oocytes and zygotes. In oocytes, 30.05% of the non-coding transcripts were associated with LTR and 69.95% were not. In zygotes, 28.65% were associated with LTR and 71.35% were not (Figure 4A). Next, we explored which ERV families these LTR-associated transcripts belong to. Statistical analysis revealed that LTR-associated transcripts in oocytes and zygotes were all from the ERV1, ERVK, ERVL, and MALR families and that most of the transcripts were from the endogenous retrovirus families ERVK and MALR (Figure 4B). Next, the lengths and expression levels of LTR-associated non-coding transcripts with coding transcripts were compared. As expected, the expression levels of LTR-associated non-coding transcripts were lower than for coding transcripts (Figure 4C). Also, the length of LTR-associated non-coding transcripts was lower than that of coding transcripts (Figure 4D).

### Implications of LTR-lncRNAs in early embryonic development in pigs

**FIGURE 5:**
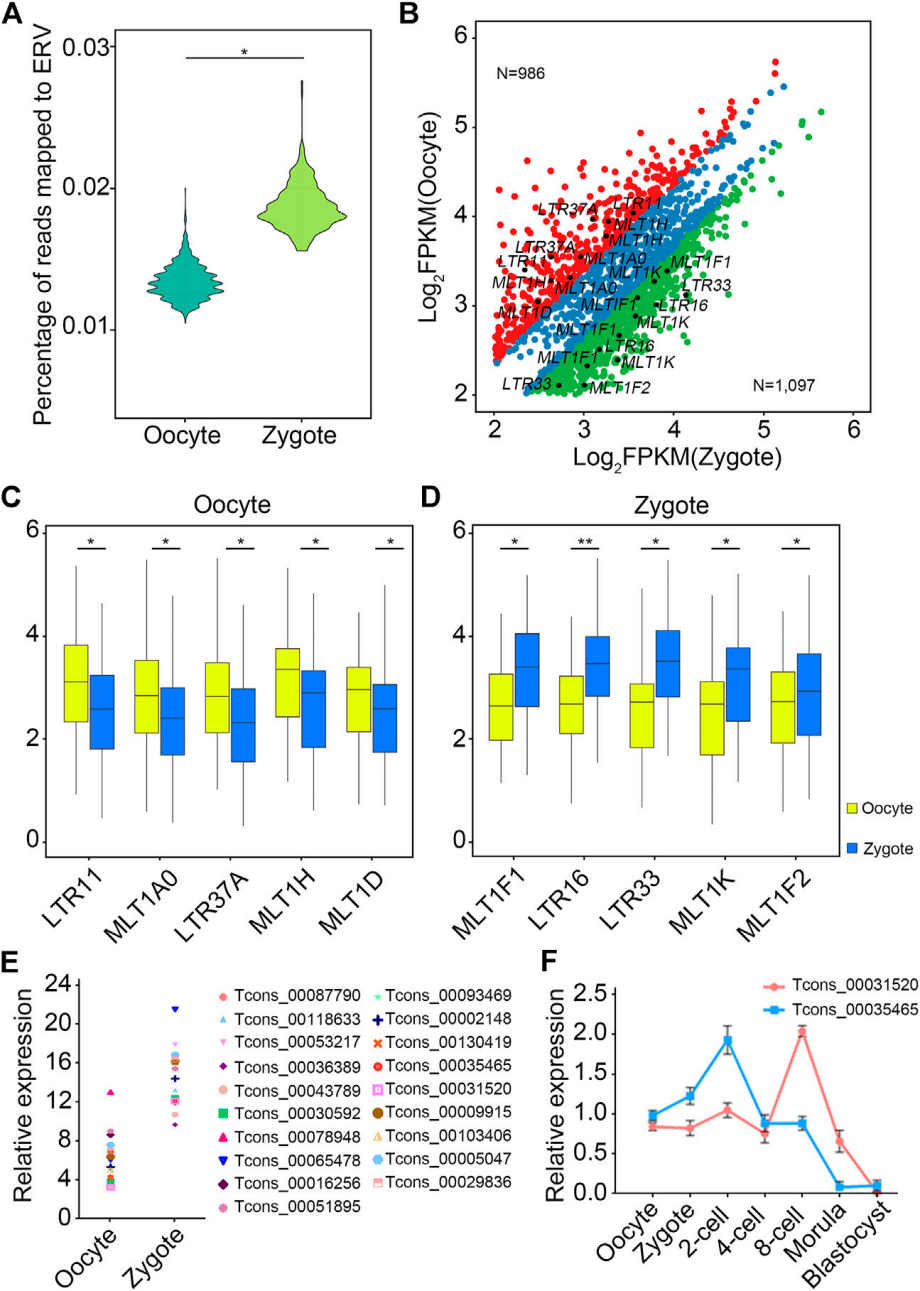
LTR-lncRNAs are functional in porcine early embryonic development. **(A)** The number of reads mapped to ERV in zygotes was higher than in oocytes. **(B)** Differential expression analysis of ERV-associated lncRNAs from oocytes and zygotes. The red dots indicate an LTR that is highly expressed in oocytes, the green dots indicate an LTR that is highly expressed in zygotes, and the blue dots indicate an LTR that has no significant difference between oocytes and zygotes. **(C,D)** The top five LTR-associated transcripts in oocyte (C) and zygote (D), respectively. **(E,F)** TCONS_00035465 showed high expression at the two-cell stage, and TCONS_00031520 showed high expression at the eight-cell stage.

Next, the expression levels of LTR-associated transcripts in oocytes and zygotes were analyzed. First, analysis showed that the number of reads that could be mapped to ERV in in zygotes was higher than in oocytes (Figure 5A). Next, differential expression analysis of ERV-associated lncRNAs from oocytes and zygotes was performed (Figure 5B), with a further screen for highly expressed ERV-associated lncRNAs in oocytes and zygotes (Figure 5B). Among all lncRNAs, five LTR-associated transcripts, LTR11, MLTA0, LTR37A, MLT1H, and MLT1D, were highly expressed in oocytes (Figure 5C), while MLT1F1, LTR16, LTR33, MLT1K, and MLT1F showed higher expression levels in zygotes (Figure 5D). Then, we focused on the highest-expressed LTR in the zygote. The 19 candidate lncRNAs from the LTR mentioned above showed higher expression levels in zygotes than in oocytes, as expected (Figure 5D). To investigate the potential regulatory roles of these 19 lncRNAs in the early embryonic development of pigs, a knockdown experiment was performed. We microinjected specific siRNAs into MII oocytes and observed embryo development at 24, 48, and 156 h after IVF. Among the 19 transcripts, two lncRNAs showed significant effects on embryo development after knockdown, namely TCONS_00035465 and TCONS_00031520 (Table 3). These results suggest that these two lncRNAs may have certain functional mechanisms during early embryonic development in pigs. Next, the expression levels of TCONS_00035465 and TCONS_00031520 at different stages were investigated. TCONS_00035465 showed high expression at the two-cell stage, and TCONS_00031520 showed high expression at the eight-cell stage (Figure 5E). We observed a significant decrease in cleavage rate after the TCONS_00035465 knockdown, suggesting that TCONS_00035465 may participate in the cleavage process. While no significant differences were detected in the cleavage rate and four-cell rate of embryos after TCONS_00031520 knockdown, we observed a significant decrease in the blastocyst rate compared with the control group (Figure 5F). This suggests that TCONS_00031520 may be involved in the development from eight-cell to blastocyst.

**TABLE 3:** The effect of lncRNA knockdown on early embryonic development of pigs.

Injected content	No. of trials	Total no. of embryos	No. of two-cell (% of $\frac{2-cell}{Total}$ )	No. of four-cell (% of $\frac{4-cell}{2-cell}$ )	No. of blastocysts (% of $\frac{Blastocysts}{2-cell}$ )
Control	3	104	70 (66.53 ± 7.33)^a^	53 (80.17 ± 4.28)^a^	25 (36.78 ± 6.10)^a^
NC	3	90	63 (69.81 ± 1.41)^a^	45 (71.33 ± 5.01)^a^	24 (39.19 ± 3.67)^a^
TCONS_00053217	3	92	59 (64.13 ± 2.12)^a^	41 (68.49 ± 5.27)^a^	17 (36.19 ± 10.14)^a^
TCONS_00036389	3	89	52 (62.09 ± 4.59)^a^	39 (74.69 ± 1.25)^a^	17 (31.94 ± 5.69)^a^
TCONS_00043789	3	97	55 (61.84 ± 5.74)^a^	42 (72.36 ± 4.16)^a^	19 (37.64 ± 4.95)^a^
TCONS_00005047	3	100	59 (59.72 ± 5.11)^a^	49 (80.28 ± 0.95)^a^	15 (30.58 ± 1.42)^a^
TCONS_00030592	3	106	69 (64.90 ± 7.03)^a^	58 (84.77 ± 7.24)^a^	20 (29.19 ± 1.14)^a^
TCONS_00002148	3	98	73 (74.99 ± 6.81)^a^	61 (81.95 ± 6.64)^a^	18 (29.31 ± 1.93)^a^
TCONS_00009915	3	105	77 (72.93 ± 4.52)^a^	58 (74.51 ± 18.30)^a^	23 (30.43 ± 3.45)^a^
TCONS_00016256	3	79	57 (70.64 ± 4.89)^a^	44 (78.65 ± 5.20)^a^	17 (29.78 ± 0.92)^a^
TCONS_00130419	3	98	79 (70.20 ± 2.53)^a^	48 (71.21 ± 9.13)^a^	21 (29.83 ± 3.87)^a^
TCONS_00035465	3	100	47 (46.89 ± 4.56)^b^	25 (52.84 ± 24.51)^a^	7 (15.06 ± 7.82)^b^
TCONS_00093469	3	99	63 (64.94 ± 7.84)^a^	44 (70.03 ± 3.59)^a^	12 (27.34 ± 5.19)^a^
TCONS_00087790	3	108	71 (66.07 ± 4.11)^a^	47 (66.30 ± 5.38)^a^	19 (26.74 ± 1.76)^a^
TCONS_00065478	3	80	50 (61.67 ± 4.91)^a^	32 (65.20 ± 6.06)^a^	15 (29.61 ± 1.78)^a^
TCONS_00103406	3	75	45 (60.91 ± 8.53)^a^	29 (64.04 ± 4.68)^a^	12 (26.38 ± 2.60)^a^
TCONS_00051895	3	103	65 (62.13 ± 8.13)^a^	46 (73.40 ± 9.51)^a^	18 (28.08 ± 2.25)^a^
TCONS_00118633	3	96	64 (67.62 ± 5.39)^a^	40 (62.22 ± 3.14)^a^	18 (28.33 ± 2.36)^a^
TCONS_00029836	3	112	58 (50.35 ± 1.15)^b^	38 (66.45 ± 11.26)^a^	10 (18.10 ± 8.46)^a^
TCONS_00078948	3	88	55 (63.21 ± 4.68)^a^	37 (68.41 ± 4.66)^a^	15 (28.14 ± 3.70)^a^
TCONS_00031520	3	96	56 (58.33 ± 12.25)^a^	50 (89.72 ± 12.10)^a^	5 (7.79 ± 5.90)^b^

Note: Different letters (between a and b) indicate significant differences (*p* < 0.05).

## Discussion

lncRNA is becoming a popular subject in scientific research. Much research has shown that lncRNAs play an important role in regulating stem cell pluripotency, reprogramming, and early embryonic development. Numerous studies have been conducted in mice, humans, and other model organisms to explore how lncRNAs regulate embryonic development and reprogramming. However, to date, research on the mechanisms of porcine preimplantation embryos and pluripotent stem cells has been extremely limited. Currently, known methods of transcribing annotations include 5′ RACE, 3′ RACE, RNA-seq, GAGE-seq, and Nano GAGE-seq (Shiraki et al., 2003; Kodzius et al., 2006; Plessy et al., 2010). RNA-seq is emerging as an attractive method to study gene expression levels in terms of reducing sequencing costs. However, RNA-seq has its own technical limitations. Due to the small number of embryonic cells and their relatively low expression levels, it is difficult to analyze lncRNA in preimplantation pig embryos. In our study, the combination of RNA-seq data and sRNA-seq data improved the incomplete annotation of the transcript, laying an important foundation for subsequent study of the effects of lncRNAs on early pig embryo development after the relatively complete transcript is obtained.

As mentioned above, it is difficult to annotate lncRNAs in early pig embryos, and studies on lncRNAs related to early pig embryo development are relatively fewer than for other species. In this study, RNA-seq and sRNA-seq data of porcine oocytes and zygotes were combined to annotate the transcripts by RSCS. We found that sRNA was involved in the assembly of more than 80% of transcripts. In addition, we used bioinformatics methods to analyze the locations of aligned sRNA and found that most sRNA was located at the 5′ and 3′ regions of the transcripts. These results indicate that the combination of RNA-seq and sRNA-seq data may be helpful in obtaining relatively complete transcript sequences, which can promote the study of the functional mechanism of lncRNA in the future.

The characteristics of the transcripts we obtained were also analyzed. Transcripts with sRNA were longer in length, with more A and G at the first base and more complete motifs than transcripts without sRNA, indicating that the transcripts annotated with small RNAs were full-length.

Some research has shown that ERV plays an important role in early embryonic development, and LTR plays an important role in the mechanism of ERV. Therefore, we explored which of the obtained transcripts were derived from LTR. LTR was found to be associated with 30% of transcripts. These LTR transcripts belong to the ERV family. We found that these transcripts are mainly derived from the ERVK and MALR families. Then, the length and expression of LTR-derived non-coding transcripts and coded transcripts were compared.

Pig is a suitable model animal for human organ donors, and there has been increasing research on early embryonic development and the pluripotency of stem cells in pigs in recent years. We believe that the study of early embryonic development in pigs is crucial for advancing medical and agricultural development. In this study, transcripts with high expression in the zygote were knocked down to observe the embryonic development level. A significant decrease in cleavage rate after TCONS_00035465 knockdown showed that TCONS_00035465 may participate in the cleavage process. While no significant differences were detected in the cleavage rate and four-cell rate of embryos after TCONS_00031520 knockdown, we observed a significant decrease in the blastocyst rate as compared with the control group. This suggests that TCONS_00031520 may be involved in the development from eight-cell to blastocyst.

In summary, we applied the RSCS to annotate augmented but high-confidence potential transcriptomes with more complete and precise contents. Taking advantage of our strategy, we identified many ERV-lncRNAs, which indicates that the set of retrotransposon functions in specific biological events has only just begun to be unraveled. We also screened out two lncRNAs that may have important functions in the early embryonic development of pigs.

## Data Availability

The original contributions presented in the study are included in the article, and further inquiries can be directed to the corresponding authors.
